# Supplementary material for: Multi-Omics Analyses Reveal the Antifungal Mechanism of Phenazine-1-Carboxylic Acid Against Pseudogymnoascus destructans
Source: J Fungi (Basel). 2025 Dec 25;12(1):16. doi: 10.3390/jof12010016 (PMC12843306; doi:10.3390/jof12010016)
Supplement: Supplementary file 1 [file jof-12-00016-s001.zip › jof-3984744-supplementary.pdf]

Multi-omics analyses reveals the antifungal mechanism of phenazine-1-carboxylic acid against *Pseudogymnoascus destructans*

Zihao Huang [1], Shaopeng Sun [1], Zhouyu Jin [1], Yantong Ji [1], Jiaqi Lu [1], Ting Xu [1], Keping Sun [2], Zhongle Li [1,3,*], Jiang Feng [1,3,*]

[1] College of Life Science, Jilin Agricultural University, Changchun 130118, China.

[2] Jilin Provincial Key Laboratory of Animal Resource Conservation and Utilization, Northeast Normal University, Changchun 130117, China.

[3] Jilin Provincial International Cooperation Key Laboratory for Biological Control of Agricultural Pests, Changchun 130118, China.

*Corresponding authors, Email: Zhongle Li, lzy1514316@126.com; Jiang Feng, fengj@nenu.edu.cn

Figure S1. Comparison of RNA-seq results of representative DEGs with RT-qPCR results.

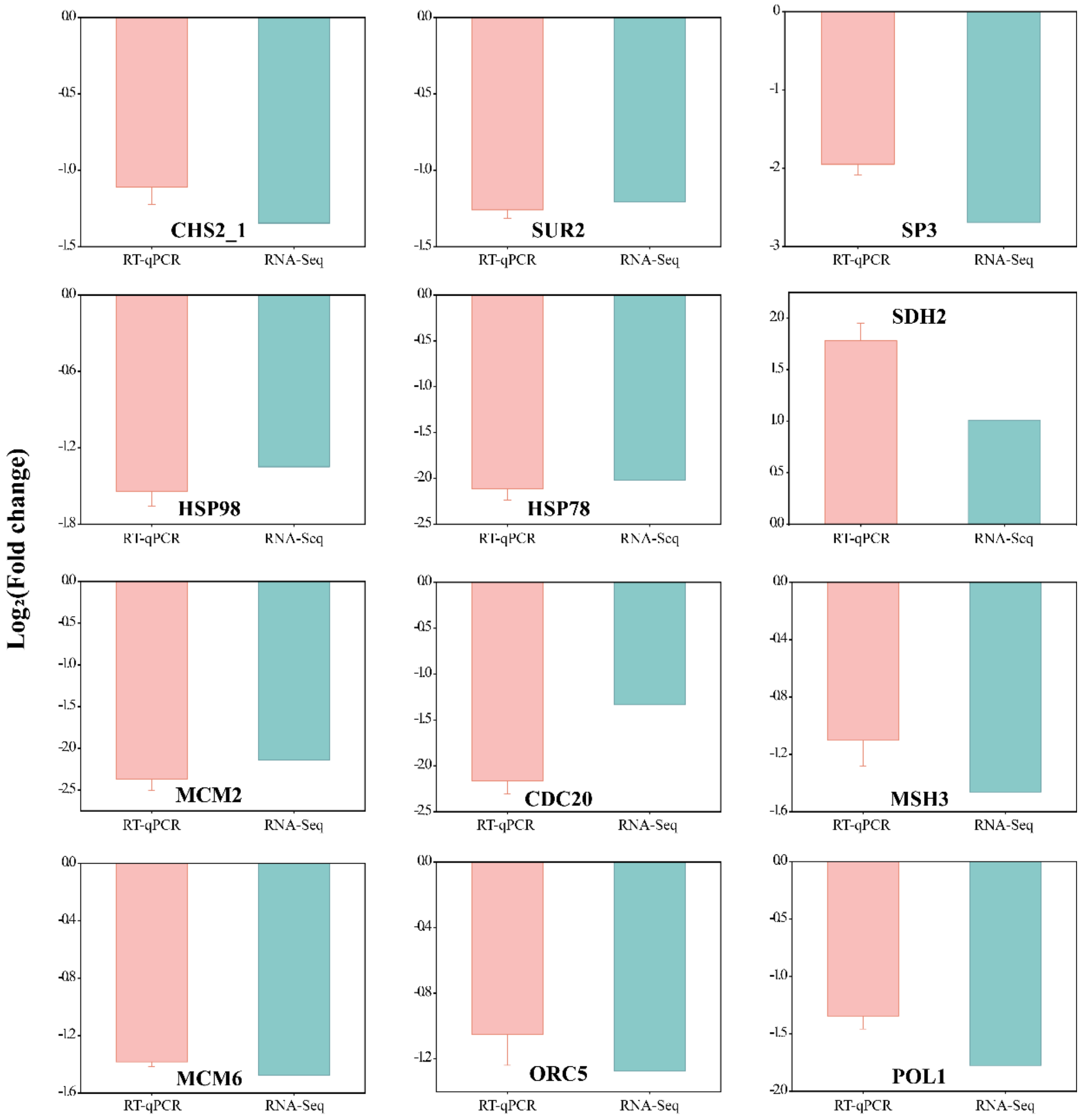

Table S1. Primers used for RT-qPCR.

Gene	Primers (5′-3′)	MW	GC Content (%)	TM (°C)	Transcript ID
CHS2_1	F: CCCCAAACTCCCTGATGGAC	6008	60	61.9	XM_024465851.1
(VC83_02183)	R: GTGGTAATATCGGCGCTCCA	6134	55	59.9	
SUR2	F: CCATTCGAGGGTTTCCTGCT	6076	55	59.9	XM_024468864.1
(VC83_05242)	R: TGGTATCCAGCGTTGTTGCT	6131	50	57.8	
SP3	F: TAACTTCATCCCCGGTTCGC	6005	55	59.9	XM_024472612.1
(VC83_09074)	R: GAGGACGTTGGAGCCCTTAC	6159	60	61.9	
HSP98	F: CATGGCGATCCTCAGCTCTT	6045	55	59.9	XM_024471698.1
(VC83_08137)	R: TTTTCTGCAGCTCGTTTGCC	6042	50	57.8	
HSP78	F: CGCGGAGATTCCAACCGATA	6112	55	59.9	XM_024464656.1
(VC83_00970)	R: TTGCCGAGTCTGGGAGAAAC	6183	55	59.9	
SDH2	F: GACCGGACCTATGATGCTCG	6119	60	61.9	XM_024466780.1
(VC83_03131)	R: GTGTGGGGGAGAGGGTAGAT	6359	60	61.9	
MCM2	F: GCCATCGCTATGACGAGGAA	6152	55	59.9	XM_024466836.1
(VC83_03188)	R: GACCGAGGAACCGTTGTCAT	6143	55	59.9	
CDC20	F: CTCTCGCCCATCACCAAACT	5943	55	59.9	XM_024464989.1
(VC83_01306)	R: ATTGGCTTGGACGACTCAGG	6174	55	59.9	
MSH3	F: AGAGCCCCTTGGATGAGGAT	6183	55	59.9	XM_024470580.1
(VC83_07000)	R: AAGAAGCTGCTCTGGGTGTC	6174	55	59.9	
MCM6	F: ATTGCCCCTCCAATGTCTGG	6045	55	59.9	XM_024469192.1
(VC83_05572)	R: GGTCGTGCTATTCCCGTAGG	6141	60	61.9	
ORC5	F: GGCGGAAGCTAAATCGCAAG	6201	55	59.9	XM_024464387.1
(VC83_00700)	R: CACCGCAGCCAAAATATCCG	6041	55	59.9	
POL1	F: CGCCGAAGCAACTGACTAGA	6121	55	59.9	XM_024469075.1
(VC83_05453)	R: GGTCGCTCATAGGTACGTCG	6150	60	61.9	
EFG1	F: AGAGGGGCATGTTGAGAAGC	6272	55	59.9	XM_024468911.1
(VC83_05289)	R: TGCGGTTCAAGGTGTAGTCC	6165	55	59.9	

F, forward primer; R, reverse primer. *EFG1*, reference gene

Table S2. Absolute electrical conductivity of PCA solutions in sterile distilled water (without mycelia) under different conditions: initial (0 h), after 24 h incubation, and after boiling.

Concentration (μg/mL)	Time (h)	Replicate 1 (μs/cm)	Replicate 2 (μs/cm)	Replicate 3 (μs/cm)	Mean ± SD
	0	3.42	3.51	3.48	3.47 ± 0.05
20	24	3.45	3.55	3.50	3.50 ± 0.05
	Boiled (30 min)	3.49	3.58	3.52	3.53 ± 0.05
	0	6.12	6.05	6.18	6.12 ± 0.07
40	24	6.15	6.10	6.22	6.16 ± 0.06
	Boiled (30 min)	6.18	6.15	6.27	6.20 ± 0.06
	0	8.35	8.28	8.41	8.35 ± 0.07
50	24	8.40	8.32	8.45	8.39 ± 0.07
	Boiled (30 min)	8.48	8.39	8.51	8.46 ± 0.06

Table S3. Overview of RNA-seq read quality.

Samples	Raw Reads	Clean Reads	N Ratio(%)	Q30 Ratio(%)	GC Ratio(%)	Total Map
CK-1	52111784	46649936	0.00%	93.73%	56.10%	92.36%
CK-2	52840738	51303312	0.00%	93.91%	56.13%	93.98%
CK-3	57034044	51303312	0.00%	94.08%	55.99%	93.76%
PCA-1	56435416	50881888	0.00%	94.14%	56.36%	93.21%
PCA-2	59807860	51101272	0.00%	93.83%	56.44%	92.58%
PCA-3	62941398	53762380	0.00%	94.03%	55.92%	89.09%

Table S4. List of Significantly Enriched KEGG Pathways of DEGs (*P* <0.05).

Pathway ID	KEGG-A-class	Pathway	Significant	Annotated	*P*-value
ko04111	Cellular Processes	Cell cycle - yeast	22/184	73/1937	3.30E-07
ko04113	Cellular Processes	Meiosis - yeast	19/184	59/1937	7.14E-07
ko03030	Genetic Information Processing	DNA replication	13/184	34/1937	5.26E-06
ko00626	Metabolism	Naphthalene degradation	6/184	10/1937	0.000103103
ko04110	Cellular Processes	Cell cycle	15/184	58/1937	0.000198916
ko00643	Metabolism	Styrene degradation	5/184	9/1937	0.000673139
ko03430	Genetic Information Processing	Mismatch repair	8/184	23/1937	0.000800173
ko00621	Metabolism	Dioxin degradation	4/184	6/1937	0.001015774
ko00624	Metabolism	Polycyclic aromatic hydrocarbon degradation	4/184	6/1937	0.001015774
ko00380	Metabolism	Tryptophan metabolism	7/184	24/1937	0.005314099
ko03440	Genetic Information Processing	Homologous recombination	6/184	20/1937	0.00844638
ko00360	Metabolism	Phenylalanine metabolism	5/184	15/1937	0.009974039
ko00350	Metabolism	Tyrosine metabolism	5/184	17/1937	0.017568363
ko00511	Metabolism	Other glycan degradation	4/184	12/1937	0.021206674
ko04068	Environmental Information Processing	FoxO signaling pathway	5/184	19/1937	0.028243006
ko00052	Metabolism	Galactose metabolism	5/184	20/1937	0.034841881
ko03450	Genetic Information Processing	Non-homologous end-joining	3/184	9/1937	0.046089284

Table S5. Significantly Enriched KEGG Pathways from GSEA of DEGs (*P* <0.05, |NES| >1).

ID	Description	Set Size	Enrichment Score	NES	*P*-value
ko00190	Oxidative phosphorylation	76	0.683785426	2.087050623	5.69E-06
ko04714	Thermogenesis	69	0.584755574	1.748693316	0.001753538
ko04723	Retrograde endocannabinoid signaling	31	0.7020714	1.805226193	0.003551727
ko03030	DNA replication	34	-0.71645936	-1.626738807	0.006780638
ko04111	Cell cycle - yeast	73	-0.566653768	-1.424225451	0.040065959
ko04110	Cell cycle	58	-0.586696018	-1.436635385	0.043589744

Table S6. List of Significantly Enriched KEGG Pathways of DEMs (*P* <0.05).

Pathway ID	KEGG-A-class	Pathway	DA Score	Significant	Annotated	*P*-value
ko01240	Metabolism	Biosynthesis of cofactors	-0.22	36/328	72/937	0.004650566
ko00740	Metabolism	Riboflavin metabolism	-0.5	5/328	6/937	0.021986276
ko00790	Metabolism	Folate biosynthesis	-0.84	5/328	6/937	0.021986276
ko01250	Metabolism	Biosynthesis of nucleotide sugars	-0.58	11/328	19/937	0.03328954

Table S7. Top 15 KEGG pathways co-enriched by transcriptomic and metabolomic analyses, ranked by the total number of enriched genes and metabolites.

Pathway ID	KEGG-A-class	Pathway	Significant (T)	Significant (M)
ko00564	Metabolism	Glycerophospholipid metabolism	5/184	74/328
ko00591	Metabolism	Linoleic acid metabolism	1/184	31/328

ko04138	Cellular Processes	Autophagy - yeast	3/184	26/328
ko04136	Cellular Processes	Autophagy - other	2/184	26/328
Ko00563	Metabolism	Glycosylphosphatidylinositol (GPI)-anchor biosynthesis	2/184	26/328
Ko00592	Metabolism	alpha-Linolenic acid metabolism	1/184	25/328
Ko04113	Cellular Processes	Meiosis - yeast	19/184	1/328
Ko02010	Metabolism	ABC transporters	1/184	16/328
Ko00350	Metabolism	Tryptophan metabolism	7/184	10/328
Ko00360	Metabolism	Phenylalanine metabolism	5/184	10/328
Ko00520	Metabolism	Amino sugar and nucleotide sugar metabolism	5/184	9/328
Ko00230	Metabolism	Purine metabolism	4/184	8/328
Ko00630	Metabolism	Glyoxylate and dicarboxylate metabolism	2/184	10/328
Ko00680	Metabolism	Methane metabolism	1/184	10/328
Ko00052	Metabolism	Galactose metabolism	5/184	6/328

Table S8. Expression of putative virulence genes in *P. destructans* following exposure to PCA. Blastx used the lowest E-value in the SwissProt database, considering only E-values < 1E-4. Genes were considered significantly different if log_2|fold change| >1 and adjusted *P*-value (Benjamini-Hochberg adjustment) < 0.05 on DESeq2 analyses.

Gene ID	Full Name	Blastx	Differentially expressed in *P. destructans*		
			FDR	Log_2FC	Result
**Proteases**					
VC83_09074	Subtilisin-like protease 3	-	0.0007	-2.7	Down
VC83_03800	Disintegrin and metalloproteinase domain-containing protein B	ADMB_ASPFU	1.99E-10	-1.3	Down
VC83_08633	Threonine aspartase 1	TASP1_HUMAN	0.009	-1.4	Down
VC83_07794	Probable aspartic-type endopeptidase opsB	OPSB_ASPOR	4.24E-21	-2.5	Down
VC83_06748	Putative aspergillopepsin A-like aspartic endopeptidase AFUA_2G15950	Y5950_ASPFU	0.001	-1.4	Down
VC83_07049	Bleomycin hydrolase	BLMH_HUMAN	3.29E-07	-1.4	Down
VC83_03174	Calpain-8	CAN8_MOUSE	1.15E-05	-2.3	Down
**Cell Wall Remodeling**					
VC83_02138	Chitin synthase 2	-	0.001	-1.3	Down
VC83_00788	Chitinase 1	CHI1_APHAL	9.57E-29	-2.5	Down
VC83_06105	Chitinase 1	CHI1_APHAL	0.0008	-2.0	Down
VC83_05476	Cell wall alpha-1,3-glucan synthase ags1	-	0.017	1.1	Up
VC83_05475	Cell wall alpha-1,3-glucan synthase ags1	-	8.30E-05	1.4	Up
VC83_01854	GPI inositol deacylase	-	2.17E-08	-1.2	Down
VC83_04980	RHO1 GDP-GTP exchange protein 2	-	0.0003	-1.2	Down
VC83_03500	Spherulin-1A	SR1A_PHYPO	2.98E-23	-2.8	Down
VC83_07867	Uncharacterized protein AFUA_6G02800	YA280_ASPFU	1.20E-08	1.5	Up
VC83_00788	Endochitinase 1	CHI1_APHAL	9.57E-29	-2.5	Down
VC83_09076	Glucan 1,3-β-glucosidase	EXG1_COCCA	6.38E-15	-2.9	Down
VC83_05292	Cell wall mannoprotein CIS3	CIS3_YEAST	1.02E-41	3.1	Up
**Ion Homeostasis**					
VC83_01014	Calcium-transporting ATPase 2	ATC2_SCHPO	3.58E-06	-1.8	Down
VC83_01092	Iron transport multicopper oxidase fetC	FETC_EPIFE	1.38E-06	1.1	Up
VC83_00191	High-affinity copper transporter ctrA2	CTRA2_ASPFU	4.86E-06	-1.5	Down
VC83_01360	Zinc-regulated transporter 1	ZRT1_CANAL	1.21E-07	-1.1	Down
VC83_01963	Laccase-1	LAC1_BOTFU	2.70E-06	-2.3	Down
VC83_07150	Iron transport multicopper oxidase FET3	FET3_GIBZE	1.73E-29	-1.5	Down
**Heat Shock Response**					
VC83_08137	Heat shock protein hsp98	-	0.02	-1.3	Down
VC83_00970	chaperone ATPase hsp78	-	0.0004	-2.0	Down
VC83_07843	hsp70 nucleotide exchange factor fes1	-	0.02	-1.3	Down
VC83_01964	Heat shock protein hsp88	HSP88_NEUCR	0.002	-1.6	Down
VC83_02553	30 kDa heat shock protein	HSP30_NEUCR	0.002	-1.2	Down
VC83_00522	Protein psi1	PSI1_SCHPO	0.004	-1.6	Down
VC83_09034	Unchar. J domain-containing protein C63.13	YCJD_SCHPO	3.49E-06	-1.0	Down
**Other**					
VC83_01624	Leptomycin B resistance protein pmd1	PMD1_SCHPO	3.54E-24	-1.8	Down

VC83_06307	Squalene epoxidase	-	0.003	-1.2	Down
VC83_03222	GTP cyclohydrolase II	-	0.02	-1.0	Down
VC83_08771	Probable transporter MCH5	MCH5_YEAST	2.49E-26	1.7	Up
